# Evaluation of the Safety and Ochratoxin A Degradation Capacity of *Pediococcus pentosaceus* as a Dietary Probiotic with Molecular Docking Approach and Pharmacokinetic Toxicity Assessment

**DOI:** 10.3390/ijms23169062

**Published:** 2022-08-13

**Authors:** Sungkwon Park, Jinsu Koo, Bosung Kim, Karthika Pushparaj, Arunkumar Malaisamy, Wen-Chao Liu, Balamuralikrishnan Balasubramanian

**Affiliations:** 1 Department of Food Science and Biotechnology, College of Life Science, Sejong University, Seoul 05006, Korea; 2 Department of Zoology, School of Biosciences, Avinashilingam Institute for Home Science and Higher Education for Women, Coimbatore 641043, India; 3 Transcription Regulation Group, International Centre for Genetic Engineering and Biotechnology (ICGEB), New Delhi 110067, India; 4 Department of Animal Science, College of Coastal Agriculture Sciences, Guangdong Ocean University, Zhanjiang 524088, China

**Keywords:** *Pediococcus* sp., probiotics, ochratoxin A, biodetoxification, toxicity assessment

## Abstract

The present study evaluated the properties and ochratoxin A (OTA) degradation capacity of the dietary probiotic *Pediococcus pentosaceus* BalaMMB-P3, isolated from a milk coagulant. The acidic tolerance of the isolate at pH 2–3 was checked with bile salts. No hemolytic activity was noted, which confirmed the nonpathogenicity of the strain. The isolate was tested in vitro for antibiotic susceptibility, enzymatic activity, bile salts hydrolase activity and antifungal activity against *Penicillium verrucosum*, *Fusarium graminearum* and *Aspergillus ochraceus*. A molecular docking-based OTA toxicity assessment was carried out for multitargeted proteins. The 16S rRNA gene-based phylogenetic assessment identified the strain as *P. pentosaceus,* and was authenticated in GenBank. The carboxylesterase and glutathione s-transferase enzymes showed active and strong interactions with esters and amide bonds, respectively. The compound exhibited carcinogenic and cytotoxicity effects at an LD_50_ value of 20 mg/kg. Furthermore, the strain showed a potent ability to reduce OTA and suggested the prospects for utilization in nutritional aspects of food.

## 1. Introduction

A vital factor and concern in food security is the presence of harmful contaminants such as mycotoxins [1], which expedite toxic effects in humans and livestock. The genera *Aspergillus*, *Penicillium* and *Fusarium* are potent producers of ochratoxin A (OTA), aflatoxin B1, deoxynivalenol and zearalenone [2]. Certainly, OTA is the most powerful amongst all other fungal toxins; moreover, the World Health Organization [3] and Food and Agriculture Organization [4] have alarmed the public that a greater risk is associated with fungal toxins than additives or residual pesticides in food safety [5]. These toxins cause genotoxicity, immunosuppression, neurotoxicity, nephrotoxicity and carcinogenesis; thus, a spectrum of research efforts has accelerated the discovery of methods for reducing the mycotoxin levels in foods [6]. Globally, chief food commodities such as cereals, fruits, spices and seeds are mainly associated with mycotoxin contamination [7]. These include fungal contamination and spoilage during the postharvest of agricultural products. The fungi spores cause hyperallergic conditions in humans, are heat resistant and easily withstand mechanical treatments such as cleaning and heating and thrive in extreme physical conditions such as high temperatures of up to 25–30 °C, a moisture content of 16–30%, and relative air humidity of 80–100% [6]. Exposure to OTA at a high concentration for a long time causes tumors in the kidney, liver toxicity, deformities and immunosuppression, leading to an increased incidence of diseases [8].

Due to the increasing importance of cereals in human and livestock diets, in recent years, much attention has focused on preventive measures for the elimination or reduction in mycotoxins. Biodetoxification through microorganisms, such as yeasts, fungi and bacteria, has been proposed as a safe strategy due to its low harmful effects in the environment, high binding affinity and toxin sequestration capacity. In particular, lactic acid bacteria (LAB) have accelerated the potential for weakening mycotoxin effects, especially OTA [9]. LAB are well known Gram-positive, acid-tolerant and immotile organism probiotics used in food bio preservation due to their active metabolites, bio protection properties and ability to use in the manufacturing of fermented products [10]. LAB with probiotic activity play a beneficial role in the human gastrointestinal tract, releasing various enzymes into the intestines and affecting digestion [11]. *Lactobacillus*, *Bifidobacterium* and *Pediococcus* genera are widely used as probiotics and are known to exert beneficial effects due to their desirable properties, including survival and colonization in the gastrointestinal passage, offering antagonistic effects against pathogenic microorganisms, tolerance against protease-rich conditions, pepsin and low pH levels of the stomach, and exhibit antimicrobial activity of bile salts in the upper part of the intestine. Moreover, antimicrobial compounds secreted from the cell wall exhibit a strong defense against fungal toxins and impede their growth [12]. The present study aimed to identify and characterize LAB from a milk coagulate and employ its potential probiotic properties in the biodetoxification of OTA. To accomplish an appropriate risk assessment, mycotoxin toxicity had to be assessed. The computational method in this research could provide precise information on the toxicokinetic and toxicity of unexplored studied mycotoxins in food and animal feed. Using the Qikrop module in the Schrodinger and ProTox-II server tool, the toxicity assessment was conducted and the molecular docking approach was performed for multitargeted enzymes.

## 2. Results

### 2.1. Molecular Characterization and Phylogenetic Analysis

**Figure 1:**
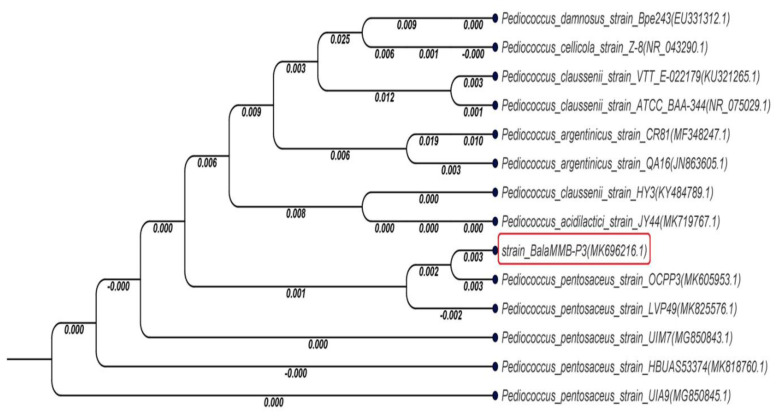
Phylogenetic tree based on 16S rDNA gene sequence showing the relationship between *Pediococcus* strains and species belonging to the genus *Pediococcus.* The highlighted red box indicated the species identified in the study.

The fragmented sequences were assembled and re-edited with sequence editor software BioEdit 7.2.5 and consensus sequences were compared with the GenBank DNA database using the basic local alignment search tool (BLAST; http://blast.ncbi.nlm.nih.gov/Blast.cgi accessed on 8 July 2022). A phylogenetic tree based on 16s rRNA gene was also constructed to determine the closest bacterial species with the neighbor-joining method, with 100 bootstrap replications performed using CLC Main workbench 8. The taxonomic position of BalaMMB-P3 was identified using 16s RNA sequences through a phylogenetic analysis. A phylogenetic analysis was carried out from NCBI blast results with a sequence alignment and tree construction carried out with CLC main workbench 8. The phylogenetic tree (Figure 1) showed that *P. pentosaceus* BalaMMB-P3 (MK696216) clustered together with other *P. pentosaecus* strains (MK696216, MK825576, MG850843, MK818760 and MG850845) with a minimum branch length. Notable differences in the branch lengths of 0.008, 0.006, 0.012 and 0.009 were observed with *P. acidilactici*, *P. argentinicus*, *P. claussenii* and *P. damnosus*, respectively.

### 2.2. Resistance to Different pH and Bile Salts

**Figure 2:**
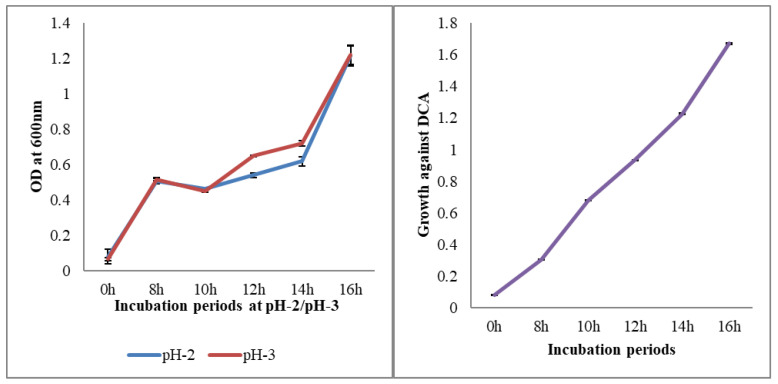
pH-resistant properties and survival ability at different pH and bile salt levels. Analysis was conducted in triplicate (n = 3).

A crucial step when choosing a probiotic strain is to assess the survivability and physiology of the bacteria under mimic conditions of the GI tract. The prospective of the selected probiotic strain should possess the ability to endure harsh conditions, such as gastric juices, high bile salt concentrations in the ileum and jejunum and effectively pass through the gut of the host. The strain *P. pentosaceus* BalaMMB-P3 exhibited substantial tolerance to low pH, determined with the plate count method. The strain exhibited resistance against low pH levels and remained unaffected in the acidic conditions. In Figure 2, an increase in bacterial growth was observed at pH 3, which showed that the strain had the ability to grow in acidic conditions. However, *P. pentosaceus* supplemented with 0.5% sodium deoxycholate (DCA) showed subtle changes in growth. This proved that the BalaMMB-P3 strain had the ability to thrive in acidic conditions due to the secretion of digestive enzymes, and could potentially be used as a probiotic to restore beneficial gut microbiota.

### 2.3. Antibiotic Sensitivity Test

Fair et al. [13] defined resistance to antibiotics as the potential for microorganisms to resist the bacteriostatic and bactericidal effects of antibiotics. Antibiotic resistance is evaluated by determining the diameter of a clear zone inhibition formed around an antibiotic disc after 24 h of incubation. The blank disc was considered as a reference to visibly distinguish the presence of clear zones. The antibiotic sensitivity pattern analysis of *P. pentosaceus* BalaMMB-P3 was tested against ten commonly used antibiotics by using the disk diffusion method. The selected isolates were resistant (≤15 mm) to ampicillin, chloramphenicol, gentamicin, kanamycin, nitrofurantoin, streptomycin, sulfamethoxazole/trimethoprim, tetracycline, cefoxitin and cefuroxime in different concentrations (Table 1).

### 2.4. Antifungal Activity of P. pentosaceus

**Figure 3:**
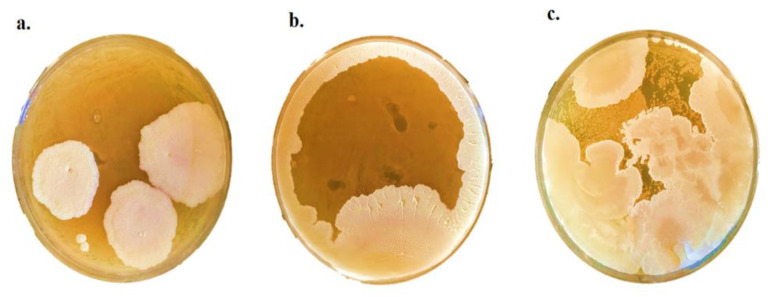
Antifungal activity of *P. pentosaceus* against *A. ochraceus* (**a**), *P. verrucosum* (**b**) and *F. graminearum* (**c**).

The antifungal activity of *P. pentosaceus* BalaMMB-P3 was analyzed against *A. ochraceus, P. verrucosum* and *F. graminearum*. The spent media of *P. pentosaceus* showed strong antifungal activity against *A. ochraceus* and *P. verrucosum,* as compared with *F. graminearum* (Figure 3). Among the two bacteria, *P. pentosaceus* exhibited potent inhibitory activity against all tested fungi as compared with the antifungal activity of *P. pentosaceus.* It indicated that they could inhibit the growth of mold that can occur in grains and cereal-based foods.

### 2.5. Hemolytic Activity

**Figure 4:**
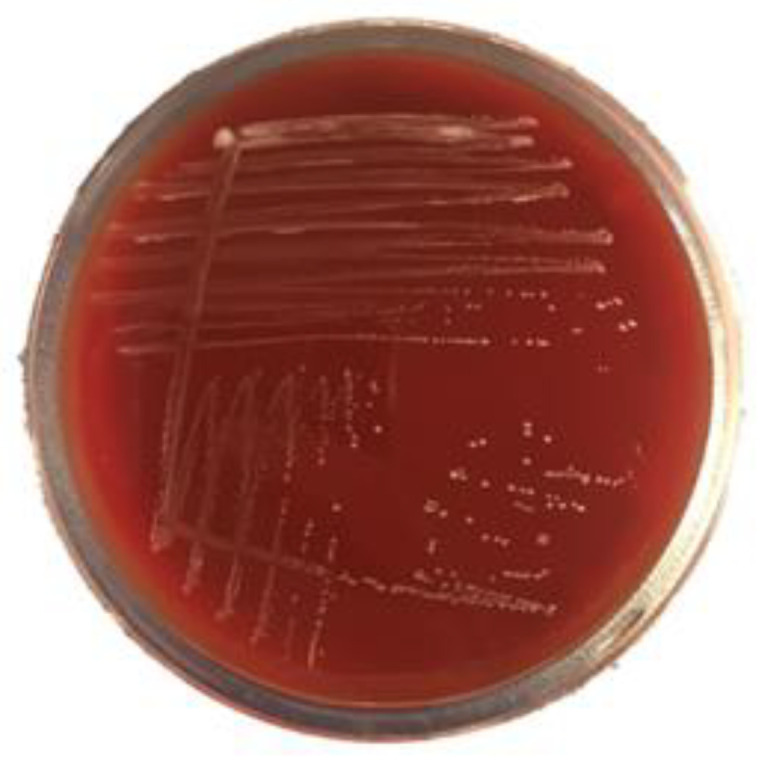
Hemolytic activity of *P. pentosaceus* BalaMMB-P3.

Hemolysis signifies the capacity of bacteria to lyse blood cells. The hemolysin accelerates the virulence of the bacterial strain which increases the rate of infection. In the present study, after incubation, white small bacterial colonies were visible, but without the clear zone surrounding the colonies. This clearly showed the absence of hemolytic activity of *P. pentosaceus* BalaMMB-P3 (Figure 4). The strain was nonvirulent, as it did not cause any lysis or infection to the cultured cells. In addition, the proliferation of cells in small colonies was noted and lysis around the zone was distinctly absent. Nonhemolytic activity is one of the vital properties for validating the probiotic potential of microorganisms, which further confirms its nonpathogenicity.

### 2.6. Enzymatic Activity

**Figure 5:**
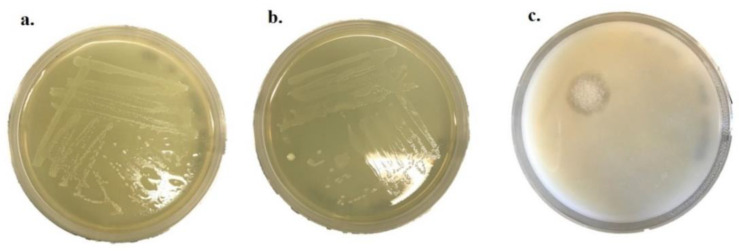
Enzymatic activities of *P. pentosaceus* in lipase agar (**a**), amylase agar (**b**) and protease agar (**c**).

The enzymatic activity of *P. pentosaceus* was assessed on amylase, protease, lipase and phytase agar (Figure 5). The halo zone diameter featuring the enzymatic activity of *P. pentosaceus* for amylase, lipase and phytase was absent. However, the enzymatic activities of protease were noted, which influenced the nutrient digestibility and augmented the role of the probiotic.

### 2.7. Detoxification of OTA

**Figure 6:**
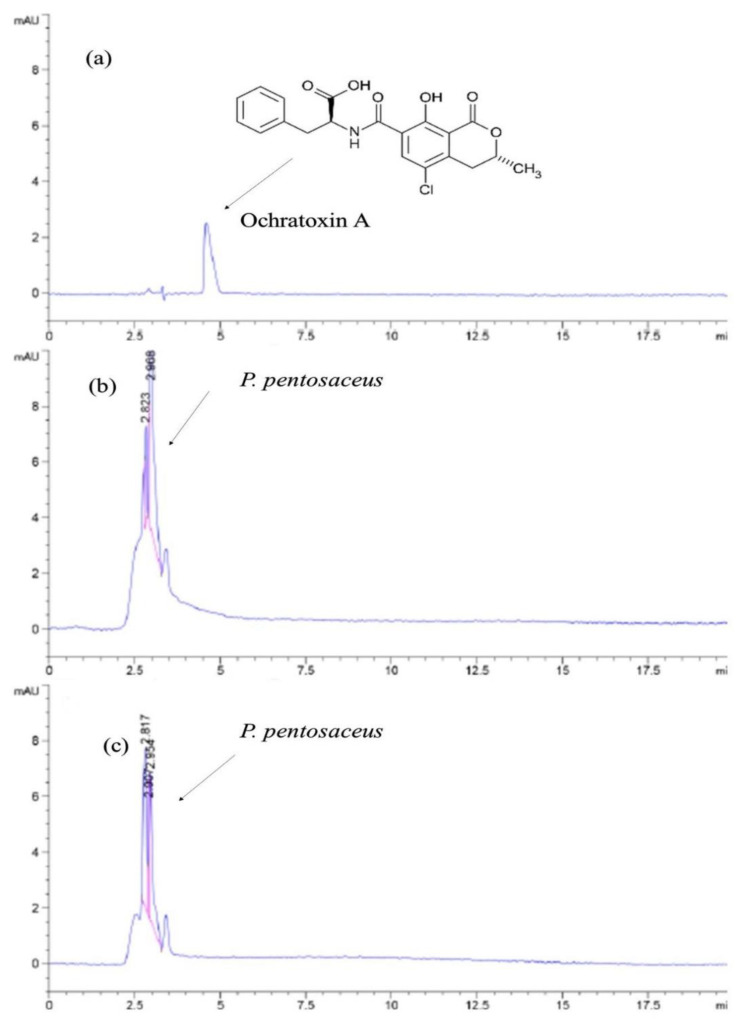
The OTA degradation (**a**) 0.8 ppm of OTA in MRS, (**b**) control (*Pediococcus pentosaceus* in MRS without OTA) and (**c**) MRS after the incubation with *P. pentosaceus* and 0.8 ppm of OTA for 24 h.

Results of the competence of LAB *P. pentosaceus* BalaMMB-P3 in detoxifying OTA concentrations in media were analyzed using HPLC, and are shown in Figure 6. After 24 h of incubation, the group with 0.8 ppm of OTA + candidate LAB culture exhibited no peak in reference to the OTA standard (0.8 ppm). In parallel, the growth of the LAB culture declined to a smaller extent; however, it was viable and exhibited sustainability even after the detoxification mechanism, which could be evidently seen in the peak of 2.072 in group c. Therefore, the removal of OTA in the media was influenced by the *Pediococcus* strain. The mechanism of correlation existed with the decline in the bacterial growth and reduction in OTA concentration.

### 2.8. Computational Approach to Detoxification

Mycotoxins, which are secondary metabolites produced by fungi, can pose major health issues to animals and humans, including monetary damages. To minimize or eliminate the mycotoxin toxicity, several ways were investigated, such as chemical methods (oxidation, hydrolysis and reduction), physical methods (thermolysis and radiation) and biological methods (enzymatic detoxification). The isocoumarin moiety in the ochratoxins was substituted with a phenylalanine group, a phenylalanine ester group and a hydroxyl group. Furthermore, the carboxyl group of the phenylalanine moiety, as well as the Cl group of the other moiety, leads one to believe in OTA toxicity.

#### 2.8.1. Molecular Docking Experiment

**Figure 7:**
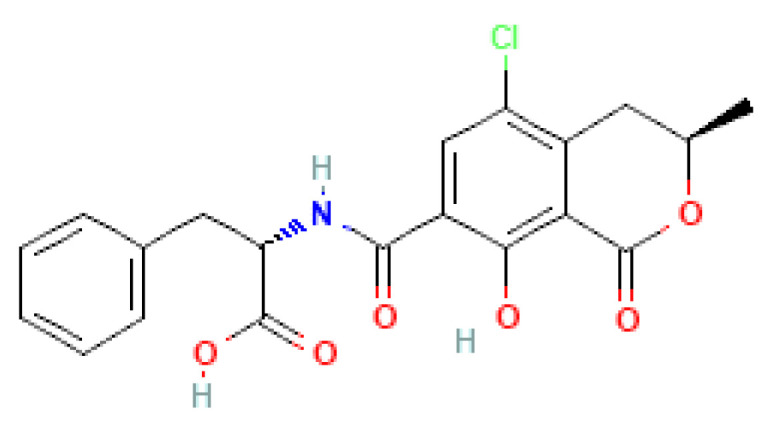
The molecular structure of OTA, the amide bond, ester, Cl and epoxide moiety at the site of detoxification.

**Figure 8:**
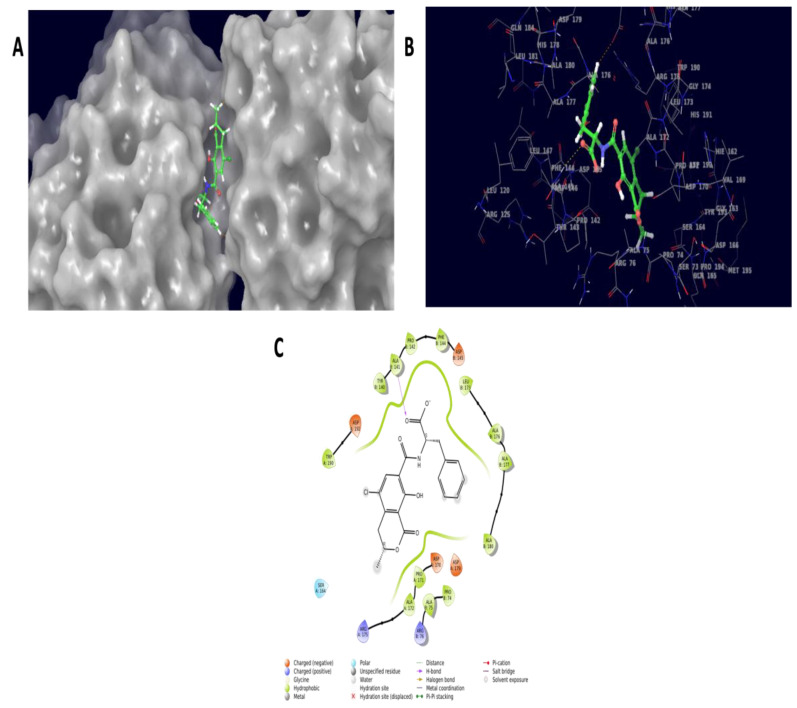
The docked complex molecule of carboxylesterase showing pocket binding sites (**A**), three-dimensional structure of interaction (**B**) and two-dimensional interaction diagram (**C**). This enzyme interacted with the ester molecule of the ochratoxin compound, which could be a possible site for degradation or detoxification mechanisms.

**Figure 9:**
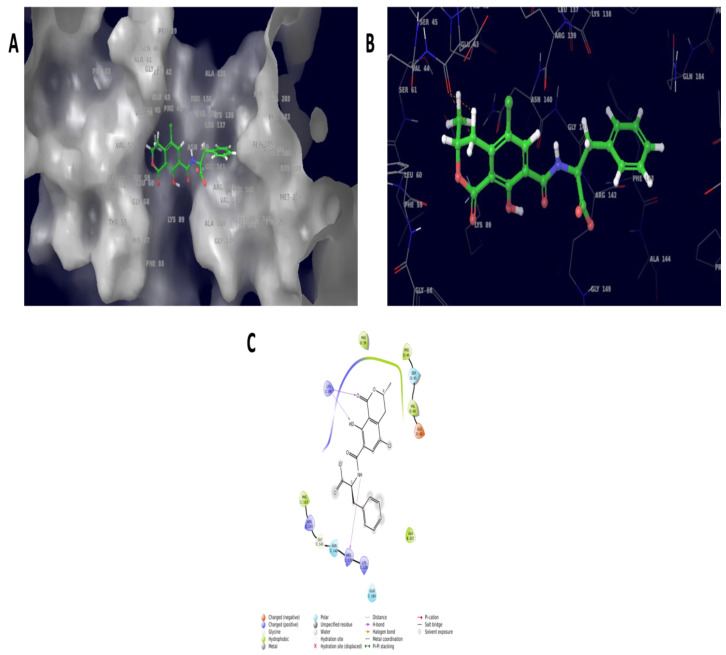
The docked complex molecule of glutathione s-transferase shows 3D pocket binding sites (**A**), the three-dimensional structure of molecular interactions (**B**) and the two-dimensional interaction diagram (**C**). These enzymes interacted with the amide bond and epoxide moiety of the ochratoxin compound, which could be a possible site for degradation or detoxification mechanisms.

Ochratoxins exploited multitarget proteins carboxypeptidase A, laccase, carboxylesterases, lactone hydrolase and glutathione s-transferase. Among them, carboxylesterases and glutathione s-transferase resulted in a docking pose with docking scores of −5.903 Kcal/mol and −2.912 Kcal/mol, respectively. Furthermore, the carboxylesterases docked with ochratoxins showed the molecular interaction of ALA141 and ASP179 with 2.7 Å and 2.32 Å distance bond lengths. Similarly, the glutathione–s-transferase complex resulted in GLU43, with 2.13 and 2.75 Å distance bond lengths. The interaction was mainly seen in the ester moiety of the OTA molecule docked against carboxylesterase. Similarly, the amide and epoxide moiety interaction was seen in the glutathione–s-transferase complex molecules. The molecular structure of OTA (Figure 7) and molecular interaction structure with 3D and 2D representations are listed in Figure 8 and Figure 9.

#### 2.8.2. Computational Toxicity Assessment

##### ADMET Analysis

The process of assessing a compound toxicity in living systems was carried out via ADMET tests utilizing the Qikrop module of the Schrodinger software. Table 2 lists the parameters such as the central nervous system (CNS) activity in a range of –2 (inactive) to +2 (active). In an aqueous solution, the number of hydrogen bonds provided (donorHB) or absorbed (accptHB) by the solute to water molecules was estimated. Furthermore, partition coefficient values for (QPlogPC16) hexadecane/gas, (QplogPoct) octanol/gas, (QplogPw) water/gas, (QplogPo/w) octanol/water, and (QPlogBB) brain/blood were anticipated. Polarizability in cubic angstroms (QPpolrz) and the IC50 value for blocking HERG K+ channels (QplogHERG) were also predicted. The solute concentrations in a saturated solution in equilibrium with the crystalline solid (S in mol dm–3), log S aqueous solubility (QPlogS) and conformation-independent aqueous solubility (CIQPlogS) were all predicted. The gut–blood barrier and blood–brain barrier were mimicked by Caco2 (QPPCaco) and MDCK (QPPMDCK) cells, respectively. Their permeability was calculated in nanometers per second. Skin permeability (QplogKp), the molecular weight of the molecule (mol MW) and binding to human serum albumin (QplogKhsa) were all estimated.

The procedure included a set of guidelines based on past knowledge, such as checking for the proper percent of human oral absorption, metabolites, rotatable bonds, logP, solubility and cell permeability values. The human oral absorption was predicted using a scale of one, two or three for low, medium and high, respectively. In addition, the scale was portrayed as a percent human oral absorption scale, which was a 0 to 100 percent scale. The prediction was determined using a quantitative multiple linear regression model. This metric usually corresponds well with human oral absorption, because they both measure the same attribute.

These parameters summarized the complex process in a biological system, in which each parameter’s value was strictly adhered to within its range of reference. This could be a drug-like substance, but, despite its bioactivity, the molecules were poisonous, as evidenced by results from carcinogenic testing [14].

The ADME results demonstrated that the compound range was inactive in the CNS, had low permeability in the gut–blood barrier, and had poor permeability in the blood–brain barrier, despite the high proportion of human oral absorption. Thus, the ADME output demonstrated that the molecules had good absorption properties, metabolic response properties and no rule violations, but had a moderate hazardous effect.

##### ProTox-II Server Analysis

Oral toxicity was predicted using the ProTox-II program based on 2D similarities and fragmentation. With an average similarity and prediction accuracy of 100 percent, the anticipated result was LD50: 20 mg/kg, which fell under the class two toxicity limit and was found to be lethal if consumed. The studies were carried out using computational methods, which showed inactive positions for all models of organ toxicity and pathways (Table 3), but active in toxicity end point models of carcinogenicity and cytotoxicity, with probabilities of 0.71 and 0.99, respectively. As a result, the compound had carcinogenicity and cytotoxicity effects that were anticipated and confirmed with the in vitro testing of carcinogenic activity with EC50 values of 0.06 M and IC50 values of 0.3 M [14].

## 3. Discussion

The contamination of food and animal feeds with OTA has been an alarming factor for decades. Research has focused on developing new and ecofriendly strategies to curtail the impact of the toxin in palatable products. A plethora of research for counteracting the mycotoxicity is available across databases. However, the isolation of an effective bacterial strain from commonly available cheap sources would be a novelty and an implication needing priority. The present study demonstrated the ability of the LAB *P. pentosaceus* BalaMMB-P3 in effectively reducing OTA toxicity in vitro. Previous studies have reported on the identification and characterization of LAB isolates *P. pentosaceus* LB44 and *Weissellaconfusa* LM85 for OTA reduction. The strains were selected based on their growth and antimicrobial activity, and identified using 16S rDNA amplification and sequencing [15]. Moreover, the biodegradation of OTA was experimented with, and reported using several LAB isolates such as *Pediococcus parvulus* from duro wines [16] and vineyard soils [17], *Brevibacterium* sp. [18], the *Cupriavidus basilensis* Őr16 strain [19], *Bacillus amyloliquefaciens* ASAG1 [20] and *Bacillus subtilis* CW 149 [21]. LAB were Gram-positive and required optimum culture conditions and enriched media for their growth; however, the isolation of LAB from naturally occurring sources is currently being studied [22] to gain vital significance. The present study is one of its kinds, and has the opportunity for logical extension on an industrial scale. Molecular identification strengthened the accurate identification and traced the taxonomic position amongst its closest neighboring species, which were determined based on sequence similarities.

*P. pentosaceus* BalaMMB-P3 exhibited high protease activity compared with other enzymes such as amylase, lipase and phytase. Similar observations were reported for LAB species *L. delbrueckiibulgaricus* CECT 4005, *Lb. paracasei* CECT and *Lb. plantarum* CECT 221, with a hydrolysis zone of more than 10 mm [23]. The protein hydrolysis in the bacteria was due to the presence of proteolytic enzymes called proteinases, which broke down the caseins into simple peptides, while the peptidases degraded the peptides and facilitated their transport across the cytoplasmic membrane. The antagonistic activity of LAB against spoilage and pathogenic microorganisms was observed as a probiotic property, which facilitated the growth of beneficial microbiomes and limited the pathogenic strains. *P. pentosaceus* BalaMMB-P3 exhibited potent antifungal activity against all tested strains. The antagonistism of LAB was due to the synthesis of nonspecific bioactive substances [24], including bacteriocins, bacteriocin-like inhibitory substances (BLISs), short-chain fatty acids (SCFAs) and hydrogen peroxide, which have a broad range of antimicrobial activities [25]. The inhibitory potential of other LAB-like strains of *L. plantarum* and *L. graminis* LabN11 was studied for antifungal activity against the ochratoxigenic strain *A. carbonarius* ANC89 and other molds [26]. The degradation of OTA was accelerated by LAB present in the cultures. In the present study, the effective removal of OTA was evidenced at a concentration of 0.8 ppm in MRS medium for 24 h. The absence of a peak denoted the removal of OTA in group c; however, a mild decline in the concentration of the microbial population was also noted. Similar results were reported on the in vitro culture of OTA with five LAB species. OTA was removed from the supernatants of LAB cultures and partially recovered from bacterial pellets. However, the degraded products of OTA were not detected in the HPLC [27]. On the other hand, *Pediococcus* strains isolated from Douro wines exhibited the potential to degrade OTA into its secondary metabolites, such as OTα. This was due to the action of peptidases that hydrolyzed the amide bond in OTA and cleaved into its metabolites. The cell wall of LAB generally possesses a high absorption capacity for toxins, which excludes the action of degradation and elimination of the toxin. In another study, the LAB *P. parvulus* UTAD 473 strain isolated from grapes degraded OTA by 50% and 90% in 6 and 19 h, respectively. Thus, LAB, particularly the *P. pentosaceus* strains, may be exploited as a potential OTA detoxifying agent to protect humas and animal health against this toxic metabolite.

Carboxylesterases are multifunctional enzymes that catalyze the hydrolysis of ester, amide and thioester bond-containing substrates. Due to their vast conformable active sites, they have broad substrate specificities, allowing them to enter a wide range of structurally varied substrates. Microbial carboxylesterases have been linked to pesticide degradation; some hydrolyze pyrethroids and bind stoichiometrically to carbamates and organophosphates [28]. A lactone moiety could be found in OTA. Lactone molecules are well-known autoregulators in both eukaryotic and prokaryotic cells. A well-known example is the quorum-sensing molecule acyl-homoserine lactone, which has been linked to biofilm formation. Biofilms are detrimental in many industrial applications; hence, high-throughput research efforts to uncover enzymes that can remove these lactone molecules have been undertaken in the past, and continue to this day. These techniques, which are typically based on metagenomics, led to the discovery of new and more efficient lactonase enzymes. Although the possible actions of these lactonases in relation to mycotoxins are unknown, scientific disciplines investigating biofilm issues may produce new microbial consortia ready to be explored for their mycotoxin-degrading capacities [29]. A chemical group referred to as the epoxide moiety has been linked to toxicity. Glutathione transferases are general detoxifying enzymes, whereas epoxide hydrolases are epoxide-detoxifying enzymes. Glutathione transferases (which degrade a variety of epoxides and require glutathione as a cofactor) are commonly found in aerobic eukaryotes and prokaryotes, such as *E. coli* and *Rhodococcus* sp. Epoxide hydrolases are found in many microorganisms, including *Flavobacterium*, *Pseudomonas*, *Corynebacterium* and *Stigmatella* species [30].

The amide bond between the isocoumarin residue and phenylalanine is hydrolyzed by a carboxypeptidase, which is the main detoxifying mechanism for OTA. Two carboxypeptidases have been linked to the breakdown of OTA: carboxypeptidase A and carboxypeptidase Y. The protease family includes the enzymes carboxypeptidase A and Y. These enzymes are particularly interesting in the realm of wastewater treatment, because they play a crucial role in the extracellular catabolism of organic matter in activated sludge. New culture-independent screening tools are being utilized to locate these enzymes, as up to 90% of bacteria found in wastewater cannot be cultured, leaving a large pool of enzymes unexplored [31].

## 4. Materials and Methods

### 4.1. Sample Collection

A standard microbial isolation described by Zhang et al. [32] with slight modifications was adopted to isolate the LAB strains from samples of a milk coagulant (curd) and cultured in MRS agar using the spread-plate technique. The cultures were incubated at 30 °C for 48 h. Colonies were isolated based on morphological differentiation and further subculture. The glycerol stock of LAB isolates was prepared, stored at −80 °C until use and they were subculture at least three times prior to the experiments.

### 4.2. Microbial Culture, Isolation of LAB and Molecular Identification

The milk coagulant was taken in a sterilized flask. Under aseptic conditions, samples were homogenized in sterile PBS (pH 7.2) to obtain an even suspension. Serial dilutions were performed on MRS agar and incubated at 30 °C for 48 h in anaerobic conditions. After the period of incubation, colonies with different morphologies on the MRS agar plate were selected and further subcultures were prepared to obtain a pure culture. The isolates from slimy colonies with different morphologies (compact, creamy, liquid and smooth) were presumptively identified as LAB. A further catalase test was performed. All reagents used were of analytical grade. The 16S rDNA of the selected isolates was amplified with PCR using primers 27F (AGT GTT TGA TCM TGG CTC AG) and 1492R (GGT TAC CTT GTT ACG ACT TC) at Tm of 55~60 °C and a GC content of 40~60%. The DNA sequencing of amplified fragments was carried out with the sequencing service of Cosmo Genetech Co., Ltd. Seoul, South Korea (http://www.cosmogenetech.com, accessed on 8 July 2022). The fragments of sequences were assembled and edited with software BioEdit 7.2.5 [Hall T (2013), BioEdit version 7.2.5 [online]; http://www.mbio.ncsu.edu/bioedit/bioedit accessed 5 December 2021], and consensus sequences were compared with those deposited in the GenBank DNA database using the basic local alignment search tool (BLAST; http://blast.ncbi.nlm.nih.gov/Blast.cgi Accessed on 8 July 2022). Sequence results were aligned with the NCBI database using the BLAST algorithm. The sequences were submitted and authenticated in GenBank. A phylogenetic tree based on 16S rRNA genes was also constructed to determine the closest bacterial species with the neighbor-joining method using Molecular Evolutionary Genetics Analysis Version (MEGA 6.0) downloaded from www.megasoftware.net accessed on 5 December 2021 [33].

### 4.3. Fungal Cultures and Preparations

Fungal strains, such as *Aspergillus ochraceus* (KACC 46484), *Penicillium verrucosum* (KACC 48466) and *Fusarium graminearum* (KACC 41045), were obtained from the Korean Agricultural Culture Collection (KACC), South Korea. One-week-old conidial spores were prepared on potato dextrose broth (PDB) and malt extract broth (MEB).

### 4.4. Evaluation of Probiotic Characteristics

#### 4.4.1. pH-Resistant

Varying pH of 2–3 of the MRS broth was prepared by adjusting the pH of growth medium with 1N HCL and 1N NaOH. Fresh cultures were inoculated onto different MRS broths and incubated for 8, 10, 12, 14 and 16 h at 37 °C. The growth curve was monitored by the measuring optical density (OD) three times at 600 nm, respectively. Analyses were performed in triplicate (n = 3).

#### 4.4.2. Bile-Salt-Resistant

The MRS broth containing 0.5% sodium deoxycholate (DCA) bile salts was prepared. Fresh cultures were inoculated onto MRS broth and incubated for 0, 12, 14 and 16 h at 30 ± 2 °C. The OD was measured three times at 600 nm and then averaged, respectively. Analyses were performed in triplicate (n = 3).

#### 4.4.3. Antibiotic Susceptibility Test

Antimicrobial susceptibility was evaluated by using an antimicrobial octa-disc (Hi-Media), followed by the method of Vlková et al. [34]. The ten chosen antibiotics, commonly used for livestock treatment, were ampicillin (10 μg), chloramphenicol (30 μg), gentamicin (10 μg), kanamycin (30 μg), nitrofurantoin (300 μg), streptomycin (10 μg), sulfamethoxazole/trimethoprim (25 μg), tetracycline (30 μg), cefoxitin (30 μg) and cefuroxime (30 μg). A fresh culture was inoculated onto MRS broth and incubated at 37 °C for 24–48 h. A fresh culture was spread on agar plates and the discs were loaded onto the plate containing the bacterial culture. The MRS agar plates were incubated for 18–48 h at 37 °C. The halo zone (diameter in mm) for each antibiotic was measured and categorized as susceptible S (≥21 mm), intermediate I (16–20 mm) and resistance R (≤15 mm), respectively [34].

#### 4.4.4. Enzymatic Activity

The enzymatic activity of the isolate was determined with the spot inoculation method in a respective enzymatic agar medium [35]. The amylase activity was examined using a medium consisting of starch (2%), agar (1.5%), yeast extract (0.7%), meat peptone (0.5%) and NaCl (0.2%). For the protease, isolates were inoculated over the medium consisting of agar (1.5%) and skim milk (1%). The phytase activity was examined using the medium composed of glucose (1.5%), NH_4_NO_3_ (0.5%), phytin (0.5%), MgSO_4_7H_2_O (0.001%) and agar (1.5%) at pH 7. Lipase activity was detected by using a medium consisting of tryptone (0.1%), yeast extract (0.5%), NaCl (0.05%), olive oil (1%), Arabic gum (1%) and agar (1.5%) and incubated at 37 ℃ for 48 h. The activity was quantified through the measurement of the halo zone (diameter in mm) surrounding each colony.

#### 4.4.5. Bile Salt Hydrolase (BSH) and Hemolytic Activity

For the BSH activity, soft MRS agar (pH 6.5, Sigma-Aldrich, St. Louis, MO, USA), bile salts (0.3% *w*/*v*, Sigma-Aldrich) and CaCl_2_ (0.375 g/L, Sigma-Aldrich) were prepared. Petri dishes with agar were incubated under an anaerobic atmosphere at 37 °C for 24–48 h. *P. pentosaceus*, which was cultured for 18 h, was inoculated on MRS agar by puncturing into the agar. The cultures containing bacterial strains were incubated at 37 °C for 72 h under an anaerobic atmosphere. Noticeable halos around the punctures indicated positive BSH activity of the strains. *Pediococcus* strains grown on MRS agar without bile salts were used as the negative control. Hemolytic activity was evaluated by streaking *P. pentosaceus* on the Columbia agar plates containing fresh blood and incubated for 24 h at 30 ± 2 °C; then, the hemolytic activity was examined. This process was repeated thrice, and an average value was noted.

### 4.5. Antifungal Property of the Isolate

The antifungal activity of the strain was evaluated with the agar diffusion method of Salminen and von Wright [36]. Petri dishes were prepared with 25 mL sterile MRS medium, and the LAB strains were streaked onto the plates and incubated at 30 °C for 24 h. After colony formation, fungal colonies were introduced onto the MRS medium and overlaid the potato dextrose agar on MRS media. The plates were incubated aerobically at 30 ± 2 °C for a week.

### 4.6. OTA Analysis of Growing Bacteria Adsorption using HPLC

The standard OTA was purchased from Glentham Life Science (UK). The methanol and acetonitrile (ANC) used were of HPLC grade supplied by Sigma-Aldrich (USA). Pure water was obtained from a Milli Q water purification system (Millipore, Billerica, MA, USA). The standard OTA was dissolved in methanol (1 mg/mL) and stored at −80 °C until use. To achieve a working standard of 1, 2, 3 and 5 ppm, the OTA solution was diluted in sterile, filtered water. A culture of the *Pediococcus* strain was incubated in 10 mL of MRS medium containing OTA (1 μg/mL) under aerobic conditions at 37 °C for 24 h. After 24 h of incubation, the bacteria were centrifuged at 3468× *g* for 10 min at 4 °C, and the bacterial supernatants were separated. The supernatant was filtered with 0.45 µm PETE Membrane 25 filters (Omnipore™) and the supernatants were collected in 2 ml vials. For the mobile phase, solvent A was water–acetonitrile (50:50, *v*/*v*). The flow rate was 0.66 mL/min, analysis time was 20 min, the injection volume was 20 µL and the fluorescence excitation and emission wavelengths were 330 nm and 460 nm, respectively.

### 4.7. Molecular Docking

The ochratoxin is a mycotoxin which has an isocoumarin moiety, carboxyl group of the phenylalanine moiety and Cl group. In order to evaluate the microorganism detoxification, it was necessary to identify the specific groups within each mycotoxin’s chemical structure that determined the harmful effects.

The OTA molecule’s three-dimensional structure was retrieved from the PubChem database in a structure data file (SDF) format; then, we imported the structure in the maestro platform and the molecule was preprocessed using a LigPrep module. The parameter was set to generate 32 stereoisomers by applying the OPLS4 force field for energy minimization. Tautomers were generated and conformations of the ligand’s orientation were assigned for future investigations [37]. The molecular targets were chosen based on their biological activity of microbial detoxification [30]. The major target proteins chosen were carboxypeptidase A (PDB ID: 1CPX), laccase (PDB ID: 6Z0K), carboxylesterases (PDB ID: 3CN9), lactone hydrolase (PDB ID: 3DHA) and glutathione s-transferase (PDB ID: 3PR8), and their three-dimensional structure was retrieved from the public dataset RCSB protein data bank in a PDB format. The target proteins were imported into the maestro platform where the preprocessing procedure was carried out. Following the assignment of a bond order, hydrogens were added, zero-order bonds were added to metals and disulfide bonds were created; selenomethionines to methionine were converted and missing side-chains were filled using the prime module; inhibitors were removed and H- bonds were optimized using the OPLS4 force field. The standard precision mode was applied for molecular docking experiments using ligand (GLIDE) docking and the resultant output was visualized in maestro using pose viewer to choose the best confirmational pose in the cluster (Schrödinger Release 2021-3: Glide, Schrödinger, LLC, New York, NY, USA, 2021).

### 4.8. In Silico Toxicity Assessment

The OTA compound molecule’s toxicity was assessed using the ADMET (absorption, distribution, metabolism and excretion) analysis using the QikProp module in Schrödinger software and the ProToX-II platform. The ADMET analysis revealed the adsorption, distribution, metabolism, excretion and toxicity of the molecule. To anticipate the pharmaceutically relevant attributes of the compounds, the physicochemical characteristics were compared to the QikProp 3.5 User Manual’s reference values (QikProp, version 3.5., Schrodinger). In the ProTox-II server, the toxicity was listed as acute toxicity (oral), organ toxicity (hepatotoxicity), toxicity end points (carcinogenicity, immunotoxicity, mutagenicity and cytotoxicity), tox21 nuclear receptor signaling pathways and stress response pathways [38,39].

### 4.9. Statistical Analysis

Values were reported as mean standard deviation (SD). All data were analyzed using R studio and Duncan’s multiple range tests. One-way analysis of variance (ANOVA) was carried out to verify significant differences among the groups at a significance level of *p* < 0.05 by using R studio (Ver. 3.3.2, Trenton, NJ, USA). All experiments were conducted in triplicate.

## 5. Conclusions

In conclusion, a novel probiotic strain, *P. pentosaceus* BalaMMB-P3, isolated from a milk coagulant, showed appreciable results in probiotic properties, such as tolerance to a low pH and bile salt sensitivity, in addition to higher enzyme activity (lipase, amylase and protease). It showed no hemolytic (γ-hemolysis) activity, which confirmed the nonpathogenicity of the strain. An in vitro experiment showed that the *P. pentosaceus* was effective in reducing OTA in low concentrations. Thus, the diverse uses of the described enzymes were fully explored in this study, and, to our knowledge, this was the first publication to provide an ochratoxin toxicity assessment computationally with a molecular docking approach.

## Figures and Tables

**Table 1:** Antibiotic sensitivity assay.

No.	Name of Antibiotics	*P. pentosaceus*
1	Ampicillin	R
2	Chloramphenicol	R
3	Streptomycin	R
4	Kanamycin	R
5	Nitrofurantoin	R
6	Gentamicin	R
7	Sulfamethoxazole/trimethoprim	R
8	Tetracycline	R
9	Cefoxitin	R
10	Cefuroxime	R

R: resistance.

**Table 2:** The ADMET profiling of ochratoxin A molecule using Qikrop module.

Parameters (Range)	Ochratoxin A
CNS (a –2 (inactive) to +2 (active) scale)	−2
mol MW (130.0–725.0)	403.818
SASA (300.0–1000)	643.884
FOSA (0.0–750)	177.286
FISA (7.0–330)	202.184
PISA (0.0–450)	203.448
WPSA (0.0–175)	60.966
donorHB (0.0–6.0)	1.25
accptHB (2.0–20)	6.5
Qppolrz (13.0–70)	38.323
QPlogPC16 (4.0–18)	12.555
QplogPoct (8.0–35)	19.694
QplogPw (4.0–45)	10.959
QplogPo/w (–2.0–6.5)	3.453
QplogS (–6.5–0.5)	−4.931
CIQPlogS (–6.5–0.5)	−5.647
QPlogHERG (concern below –5)	−3.465
QPPCaco (<25 poor; >500 great)	38.85
QplogBB (–3.0–1.2)	−1.611
QPPMDCK (<25 poor; >500 great)	40.38
QplogKp (–8.0––1.0)	−3.953
#Metab (1–8)	4
QplogKhsa (−1.5–1.5)	0.087
Human oral absorption (1–3)	3
Percent human oral absorption (above 80% was high; below 25% was poor)	75.612
Rule of five (maximum was 4)	0
Rule of three (maximum was 3)	0

**Table 3:** Toxicity assessment of ochratoxin A calculated using ProTox-II web-based server.

Classification	Target	Prediction
Organ toxicity	Hepatotoxicity	Inactive
Toxicity end points	Carcinogenicity	Active
Toxicity end points	Immunotoxicity	Inactive
Toxicity end points	Mutagenicity	Inactive
Toxicity end points	Cytotoxicity	Active
Tox21-nuclear receptor signaling pathways	Aryl hydrocarbon receptor	Inactive
Tox21-nuclear receptor signaling pathways	Androgen receptor	Inactive
Tox21-nuclear receptor signaling pathways	Androgen receptor ligand-binding domain	Inactive
Tox21-nuclear receptor signaling pathways	Aromatase	Inactive
Tox21-nuclear receptor signaling pathways	Estrogen receptor alpha	Inactive
Tox21-nuclear receptor signaling pathways	Estrogen receptor ligand-binding domain	Inactive
Tox21-nuclear receptor signaling pathways	Peroxisome proliferator-activated receptor gamma	Inactive
Tox21-stress response pathways	Nuclear factor (erythroid-derived 2)-like 2/antioxidant-responsive element	Inactive
Tox21-stress response pathways	Heat shock factor response element	Inactive
Tox21-stress response pathways	Mitochondrial membrane potential	Inactive
Tox21-stress response pathways	Phosphoprotein (tumor suppressor) p53	Inactive
Tox21-stress response pathways	ATPase family AAA domain-containing protein 5	Inactive

## Data Availability

The data presented in this study are available on request from the corresponding authors.
